# Growth Hormone (GH) and Rehabilitation Promoted Distal Innervation in a Child Affected by Caudal Regression Syndrome

**DOI:** 10.3390/ijms18010230

**Published:** 2017-01-23

**Authors:** Jesús Devesa, Alba Alonso, Natalia López, José García, Carlos I. Puell, Tamara Pablos, Pablo Devesa

**Affiliations:** 1 Scientific Direction, Medical Center Foltra, 15886 Teo, Spain; 2 Children Physiotherapy, Medical Center Foltra, 15886 Teo, Spain; alba.fisioterapia@foltra.org (A.A.); natalia.fisioterapia@foltra.org (N.L.); 3 Adults Physiotherapy, Medical Center Foltra, 15886 Teo, Spain; jose.fisioterapia@foltra.org; 4 Physical Medicine and Rehabilitation, Medical Center Foltra, 15886 Teo, Spain; cipuell@foltra.org; 5 Neurology, Medical Center Foltra, 15886 Teo, Spain; tpablos@foltra.org; 6 Research and Development, Medical Center Foltra, 15886 Teo, Spain; pdevesap@foltra.org

**Keywords:** GH, syndrome of caudal regression, sacral agenesis, physiotherapy, neurogenic bladder, flaccid paraplegia

## Abstract

Caudal regression syndrome (CRS) is a malformation occurring during the fetal period and mainly characterized by an incomplete development of the spinal cord (SC), which is often accompanied by other developmental anomalies. We studied a 9-month old child with CRS who presented interruption of the SC at the L2–L3 level, sacral agenesis, a lack of innervation of the inferior limbs (flaccid paraplegia), and neurogenic bladder and bowel. Given the known positive effects of growth hormone (GH) on neural stem cells (NSCs), we treated him with GH and rehabilitation, trying to induce recovery from the aforementioned sequelae. The Gross Motor Function Test (GMFM)-88 test score was 12.31%. After a blood analysis, GH treatment (0.3 mg/day, 5 days/week, during 3 months and then 15 days without GH) and rehabilitation commenced. This protocol was followed for 5 years, the last GH dose being 1 mg/day. Blood analysis and physical exams were performed every 3 months initially and then every 6 months. Six months after commencing the treatment the GMFM-88 score increased to 39.48%. Responses to sensitive stimuli appeared in most of the territories explored; 18 months later sensitive innervation was complete and the patient moved all muscles over the knees and controlled his sphincters. Three years later he began to walk with crutches, there was plantar flexion, and the GMFM-88 score was 78.48%. In summary, GH plus rehabilitation may be useful for innervating distal areas below the level of the incomplete spinal cord in CRS. It is likely that GH acted on the ependymal SC NSCs, as the hormone does in the neurogenic niches of the brain, and rehabilitation helped to achieve practically full functionality.

## 1. Introduction

In addition to the incomplete development of the spinal cord (SC), many different abnormalities may also appear in infants with caudal regression syndrome. Among them are urological abnormalities such as renal agenesis and neurogenic bladder, tethered-cord, sacral agenesis, lipomyelomeningocele, anorectal atresia, orthopaedic deformities, and even cardiac malformations [1]. While it is likely that most of these and other abnormalities observed in the syndrome occur as a consequence of incomplete SC development during the fetal period, some of them may be the consequence of a genetic polymalformative syndrome. This would explain the wide spectrum of clinical presentations of caudal regression syndrome (CRS).

This syndrome was first described by Duhamel in 1961 [2], as an alteration in the formation of the caudal region. In fact, this developmental abnormality has been related to neurulation alterations during the first 28 days of fetal life or to malformations occurring during the fetal differentiation phase [3]. However, the later may be a consequence of the former, since the expression of genes involved in fetal development occurs in a progressive and sequential manner. This would agree with the first description by Duhamel [2], indicating that the syndrome can present a wide spectrum of malformations, being the siren monstrosity and malformations of the anal region, both of which are extremes (maximal and minimal, respectively).

The exact cause of CRS is still unknown. It is observed in 2 live births per 100.000 newborns; however, its incidence is 150-fold higher when uncontrolled gestational diabetes exists [4,5]. No clear explanation has been given for this finding.

Apart from maternal gestational diabetes, a number of factors have been suggested to play a role in the occurrence of CRS. Among them are the mother’s alcohol intake, retinoic acid, a deficient supply of oxygen to the fetus, and putative amino acids imbalances, but no evidence exists about the possible involvement of any of them in the pathogenesis of the syndrome. Therefore, genetic reasons seem to be the most possible causes.

During fetal development, growth hormone (GH) and insulin-like growth factors (IGF-I and II) play a key role, as we recently described [6]. Fetal GH seems not to be responsible for fetal growth; most likely the hormone is involved in the developmental program of fetal tissues and organs [6]. In turn, IGFs and Insulin would be responsible for growth but also for factors involved in the developmental program of the fetus [6], in which IGF-II is mainly involved.

Apart from its effects on brain repair after an injury [7,8,9,10,11,12], we first reported that GH administration regenerated the transected sciatic nerve in rats [13], a finding also reported by other authors [14,15]. Unpublished data from our group also indicate that GH and rehabilitation improve the sensitive and motor functions of patients with SC injuries below the level of the spine, at least in ASIA (American Spinal Cord Injury Association) B and C patients [16].

On this basis, we decided to follow a similar treatment in a 9-month old child with CRS. His SC development had been interrupted at the L2–L3 level; there was sacral agenesis and right renal agenesis, neurogenic bladder and bowel, and a lack of innervation of the inferior limbs. After five years of treatment, most of the nerve alterations have been corrected, including control of his sphincters, and the child is able to walk with the help of crutches. This is the first case in which CRS (but not renal and sacral agenesis) has been corrected at the nervous level, despite it having been previously considered that, since the primary pathology is irreversible, treatment of this syndrome is only supportive [4].

## 2. Results

The patient commenced physiotherapy for rehabilitation early in his life; however no significant results had been achieved at the time that he was admitted at our center.

### 2.1. Physiotherapy

At admission, the score in the Gross Motor Function Test (GMFM)-88 test was 12.31%, due to the fact that the patient was unable to reach any punctuation in dimensions C, D, and E of this test. The Asshworth scale indicated that no spasticity existed (0/0 and 0/0, both hemibodies).

Four months after commencing with GH administration and rehabilitation, sensitivity to painful stimulation was detected at the L4, L5, and S1 levels, indicating that, despite the SC being interrupted at L2–L3, sensitive innervation was beginning to develop below the level of the lesion.

Six months after commencing the treatment, the total GMFM-88 score increased to 39.48%. This clear increase was mainly due to improvements in dimensions A and B of the test, while the score remained unchanged in the other dimensions of it (0 points in every item), with the only exception being item 38 in dimension C, in which the patient reached a maximum punctuation of 3 since he was able to crawl forward 1.80 meters. At this time, the physical evaluation (ASIA score) indicated that the patient had significantly improved in sensitivity and motor functions. There were responses to sensitive stimuli in the quadriceps, ischio-tibialis, tibiales and peroneal muscles, gastrocnemius, and feet. In addition, the patient reacted to pressor stimuli in the first phalanx of both feet but not in the other phalanges. Interestingly, an important improvement was observed in the amplitude of the movement of his knees, beginning to be able to make a small active flexion of them.

One year later, evoked osteo-tendon reflexes were present in the patellar and Achilles tendons (mild and weak responsiveness, respectively).

**Figure 1:**
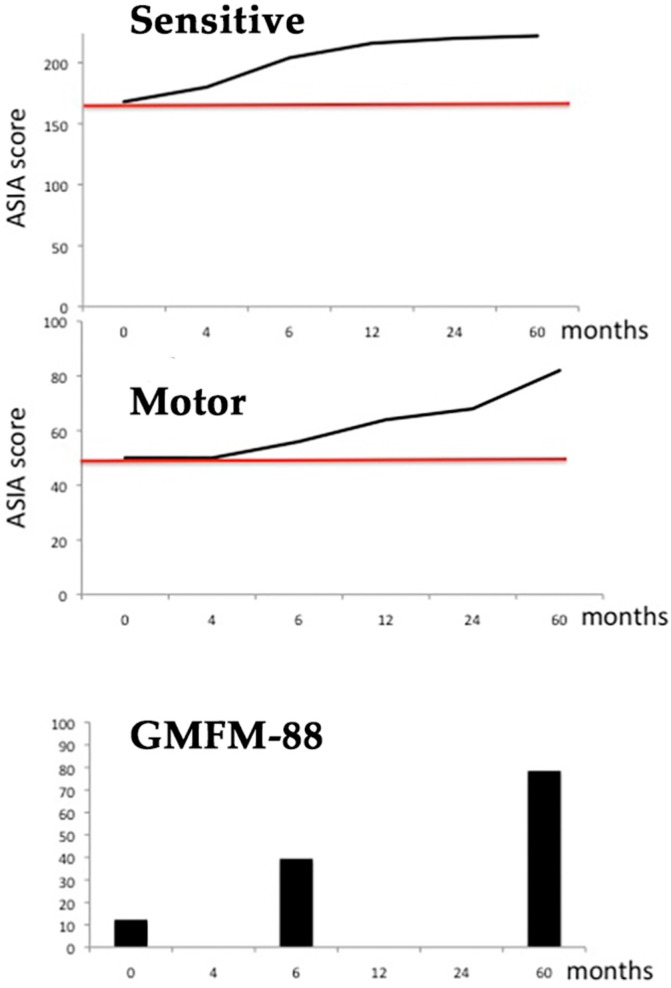
Graphic representation of the results obtained. **Upper** graph: Sensitive evolution (pin prick score + light touch score; left + right hemibodies) according to the ASIA (American Spinal Injury Association) scale. Maximal score (y axis): 224. The red line delimits the starting point (168), since no abnormalities existed above L3; **Middle** graph: Motor evolution according to the ASIA scale. Maximal score (y axis): 100. The red line delimits the starting point (50), since no abnormalities existed above L3; **Lower** graph: Evolution of the Gross Motor Function Test (GMFM)-88 test. Note that the intervals in the x-axis only indicate the age (in months) at which the scores were registered in each graph once the treatment commenced (month 0).

A new control, performed two years after commencing with GH and rehabilitation, showed that sensitive innervation was complete (Figure 1 and Movie 2), while at the motor level the patient voluntarily moved his quadriceps, ischio-tibialis, adductors, and abductors but still not any muscle below the knee. He began to walk with the help of a walker (Movie 3) and he was able to ride on a tricycle (Movie 4). His gastrocnemius muscles were still atrophic, but the Achilles reflex was clearly evoked. His legs and feet were in external rotation, most likely due to the existence of hip luxation.

No cardiac or respiratory problems existed during the treatment period.

Very importantly, the child achieved complete voluntary control of his sphincters, although urinary urgency sometimes appeared.

With regard to his left kidney problem, studies performed in two different hospitals indicated that no vesicoureteral reflux already existed.

**Figure 2:**
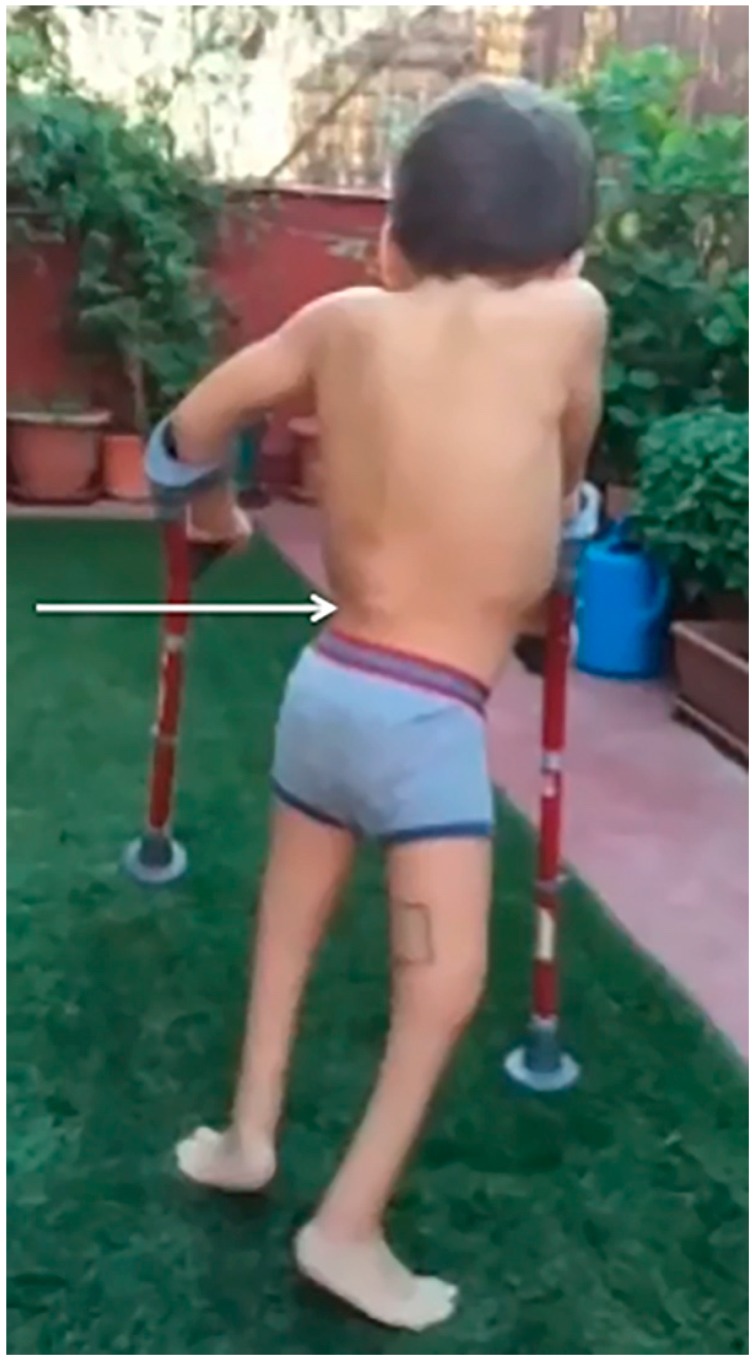
The patient is walking with the help of canes.

One year later the patient was walking with the help of crutches (Figure 2).

Three years after commencing the treatment, the patient was able to walk with crutches, although the lack of the sacrum produced a flexion of the hips while walking. Note that the muscles of the legs still lacked development, while there was an increase in the mass of the quadriceps and ischio-tibialis. Clubfoot persists. The white arrow shows where the vertebral column ends, as indicated by a bulging bone in the back.

The GH dose was increased to 0.8 mg/day, and melatonin (50 mg/day, orally, before going to bed) was prescribed to counteract a possible increased production of oxygen free radicals due to the physical effort of walking without the support of the sacrum and in the presence of hip luxation [17,18,19].

The last control, carried out 5 years after commencing with the combined treatment of GH and rehabilitation, showed that the muscles of the legs had begun to develop, mainly on the right side, and also that the three middle toes of the right foot were able to make flexion and extension movements. The left leg was about 2 cm shorter than the right. Both feet have clearly changed their appearance since the beginning of the treatment, although the left foot looked more hypotrophic than the right. However, with both feet, the patient was able to make plantar flexions against resistance and a weak dorsiflexion (Movie 5).

The height of the child is now within normal percentiles (p15) and the GH dose is 1 mg/day (5 days/week). Melatonin continued to be given at a daily dose of 50 mg.

**Figure 3:**
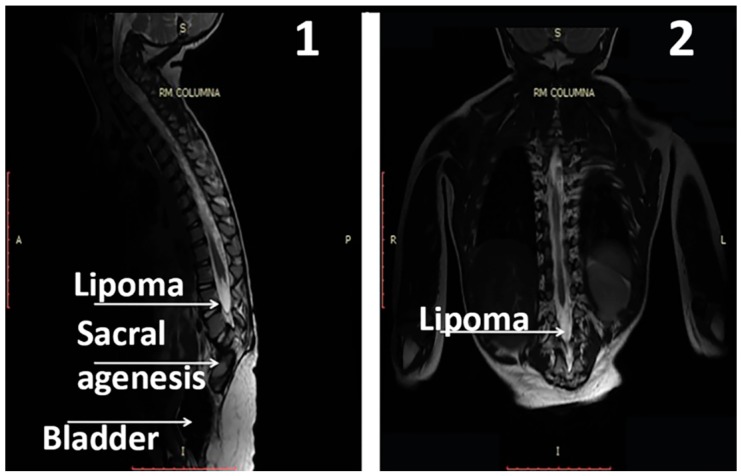
MRI performed at age 3-years old. (**1**) and (**2**) show sagittal and antero-posterior images (respectively) of the spinal cord. In 1, sacral agenesis and the hypoplastic L3 vertebra can be clearly seen (white arrow). In both images, a small lipoma can be seen in the lower part of the conus medullaris. As the images show, the SC was normal in its diameter and morphology, but a small hydrosyringomyelia can be seen. In (**2**), an asymetry between the right and left hip can be observed. A: anterior; P: posterior; S: superior; I: inferior; R: right side; L: left side.

In summary, after 5 years of treatment, significant sensorial and motor improvements had been reached; the last GMFM-88 test performed revealed a score of 78.48% (maximum = 100). This means that, despite the SC having been interrupted at the L2–L3 level (Figure 3), a significant innervation occurred below the level of the lesion, as the ASIA scores also revealed. These results are schematized in the Figure 1.

Voluntary control of his sphincters persists, but the parents were informed that a grade II vesicoureteral reflux had been detected again in the left kidney.

### 2.2. Imaging Exams

**Figure 4:**
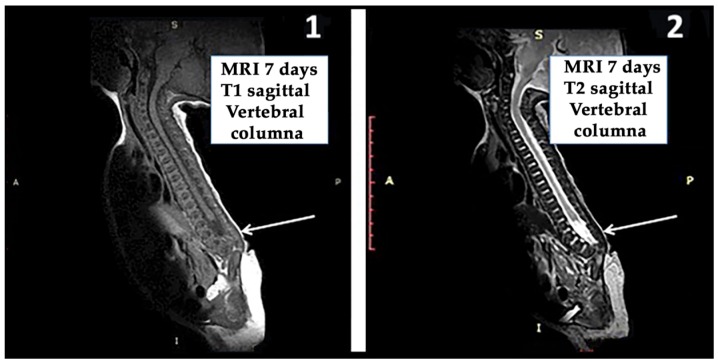
Magnetic resonance imaging (MRI) of the vertebral column MRI performed at 7-days of age. (**1**) The white arrow shows that the vertebral column development had been interrupted at the L2–L3 level and that the spinal cord was tethered; (**2**) A small lipoma can be seen at the end of the conus medullaris (arrow); there was sacral agenesis too. A: anterior; P: posterior; S: superior; I: Inferior. T1, the time when 63% of the longitudinal magnetization has recovered; T2, the time when 63% of the transverse magnetization has decayed.

A magnetic resonance imaging (MRI) study of the vertebral column, carried out 7-days after birth, showed that the vertebral development had been interrupted at the L2–L3 level; the SC was tethered at the L3 level and that there was sacral agenesis and right renal agenesis (Figure 4).

At 21-days of age, an abdominal ultrasound study showed luxation of both hips with an incomplete development of both acetabulae.

At 13-months of age, that is 4-months after commencing with GH treatment and rehabilitation, a new MRI study indicated that no changes had occurred with regard to the first study carried out 7-days after birth. The study indicated agenesis of the sacrum and of lumbar vertebral bodies L4 and L5. L3 was reduced to a simple vestige; iliac bones were articulated in the midline and both femoral heads were luxated. The spinal canal terminated at the L3 level, and a tissue with fat intensity, most likely a lipoma, could be observed in the bottom of the spinal canal. The conus medullaris ended at the level of the thoracic T12 vertebra, and both in the conus medullaris and in the total SC a prominent central ependymal canal could be observed. This ependymal canal was enlarged in the region of the conus medullaris and in the cervical SC (data not shown).

At age 3-years, a new MRI study of the SC indicated that there was no modification in the findings observed in the previous study. That is, there was agenesis of the sacrum and of vertebral bodies L4 and L5 with a hypoplastic L3, where the spinal canal ended. The conus medullaris ended at the level of the T12 vertebra and, in its lower and posterior part, presented a small lipoma, most likely corresponding to a fatty terminal filum. The SC was normal in its diameter and morphology, showing a minimal enlargement of the central ependymal canal in the lower cervical and dorsal segments; however, as reported by the radiologist, this enlargement was lower than in the previous study (Figure 3).

**Figure 5:**
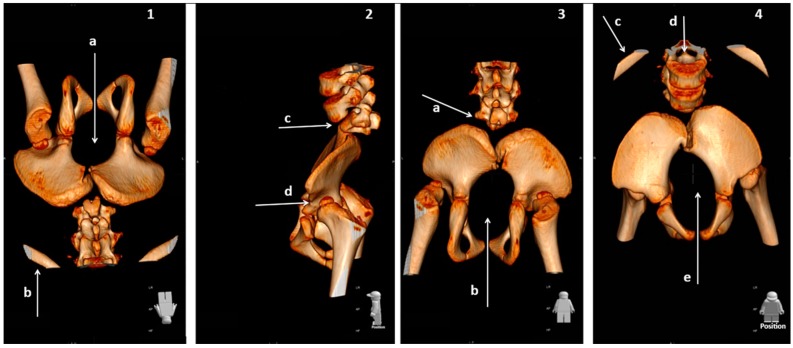
3D reconstruction of a CT-SCAN showing the vertebral and hip abnormalities. (**1**) Inverted position for a better view of the hypoplastic L3 vertebra. The arrow marked “a” shows the sacral agenesis, while arrow b indicates the 12th rib (to show where the lumbar column begins); (**2**) In this sagittal view, arrow c shows the hypoplastic L3 vertebra and arrow d shows the lack of articular congruence between the head of the femur and the abnormal left hip acetabulum; (**3**) Posterior view, in which arrow a shows the abnormal L3 and arrow b shows the sacral agenesis. Notice too the iliac bones articulated in the midline, the rotation of the hips, the incomplete development of both acetabulae, and the luxated femoral heads; (**4**) Oblique view for seeing the spinal cord (arrow d). Arrow c shows the 12th rib and arrow e shows the sacral agenesis. Note, again, the articulation of the iliac bones and the rotation of the left hip.

At 4-years old, a 3D reconstruction of a CT-SCAN allowed us to clearly see the abnormalities that have occurred during the development of the vertebral column and SC. As Figure 5 shows, vertebral development had been interrupted at the L2–L3 level. The sacrum did not exist, the iliac bones were articulated in the midline, the hips were rotated, the development of both acetabulae had been incomplete, and both femoral heads were luxated.

A new MRI study of the SC, recently carried out, showed that there was no modification in the findings observed when the child was 3-years old (data not shown).

Movie 6 shows the motor evolution of the child before he began to walk with crutches.

### 2.3. Blood Analysis

The prre-treatment blood analysis was practically normal, except for a slightly elevated plasma creatinine value (0.51 mg/dL; normal range: 0.2–0.49 mg/dL) and a low plasma IGF-I value (48 ng/mL; normal range for the age: 50–354 ng/mL), while the insulin like growth factor binding protein 3 (IGFBP3) was normal (2.4 µg/mL; normal range: 0.7–3.9 µg/mL). Erythrocytes and Hb were present in normal values, despite the serum iron being low (28 µg/dL; normal range: 40–100 µg/dL). The plasma proteins were within normal values. Plasma thyroid-stimulating hormone (TSH) was normal (3.02 µUI/mL), as was free thyroxine (fT4, 1.2 ng/dL); plasma cortisol at 8 a.m. was also normal (18 µg/dL).

Given the pathology and the age of the patient, we did not perform any provocative tests for analyzing pituitary GH secretion, despite the low height and low plasma IGF-I values.

The blood analysis carried out after 3-months of GH treatment indicated that the slight abnormalities observed in the pre-treatment study had disappeared. Plasma creatinine and IGF-I were now within normal values (0.40 mg/dL and 146 ng/mL, respectively).

Subsequent blood analysis carried out throughout the treatment always showed normal values in all the parameters analyzed. Plasma creatinine ranged between 0.3 and 0.4 mg/dL, and plasma IGF-I reached a maximal value of 254 ng/mL, while IGFBP3 oscillated around 3.5–4 µg/mL.

Treatment with GH and melatonin did not cause any adverse side effects.

## 3. Discussion

In this study, we analyze the results achieved in a child born with CRS and sacral agenesis who was treated with GH and rehabilitation. To our knowledge, this is the first report about an almost complete development of innervation below the level of the SC affectation in this rare congenital syndrome, a fact that we have to attribute to the combined treatment with GH and rehabilitation that the child has received since he was 9-months old. Since the primary pathology has been reported as irreversible, treatments for CRS have been considered to be only supportive [4]. We know that this is only one case among the wide spectrum of abnormalities that can appear associated with this syndrome, and it is possible that other types of CRS or children treated at more advanced ages, might not have an evolution as favorable as our patient showed, but, in any case, GH treatment and rehabilitation resulted in promoting sensitive and motor innervation and bowel and bladder control.

As expected, the treatment used could not recover the lost sacrum bone nor the renal agenesis or orthopedic anomalies, but, in our opinion, most of these could be solved surgically in the coming years. Hence, the quality of life of the patient was improved considerably with regard to the initial prognosis.

Despite the successful results obtained with the combined treatment of GH and rehabilitation, we cannot know the exact role that the hormone played. However, it is unlikely that rehabilitation alone would have produced these results. In fact, before receiving GH, the child had been treated exclusively with rehabilitation without any improvement. Moreover, the rehabilitation commenced early after birth, a period of time during which high plasticity exists, at least at the nervous system level. This is consistent with a number of reports that describe the treatment of this syndrome as merely supportive, addressed to correct orthopedic and other abnormalities (cardiac, gastrointestinal, vertebral, respiratory, etc), if they exist, in order to improve the patient’s quality of life [1,3,4,5,20,21,22,23].

At this point, in order to try to understand how GH acted in this case, we should review the possible causes of the syndrome.

The existence of uncontrolled maternal diabetes and mutations in the homeobox gene HBLX9, also expressed in the pancreas, have been reported to be related with the development of CRS [21,23,24,25,26]. However, this was not the case in our patient. As described in the introduction, his mother did not have diabetes and the genetic studies of the child were normal. On the other hand, although the incidence of the syndrome increases to 1 in 350 when uncontrolled gestational diabetes exists [4,5], it has been found that only 16% to 22% of the mothers of CRS patients have diabetes; therefore it seems that the syndrome is not specific to diabetes, at least in humans.

We and others have demonstrated [13,14,15] in rats that early GH treatment is able to recover sciatic nerve transection, increasing the number of Schwann cells that produce myelin, promoting axonal regeneration and muscle reinnervation; consequently, the affected muscles recover their trophism. This, and our unpublished studies in patients with SC injuries [16], indicates that GH is able to promote peripheral axonal growth, and this might explain the effect of the hormone on the innervation observed in our CSR patient. However, a clear difference exists between repairing an injured nerve in a previously innervated zone and innervating a large area that had never received nervous stimulation, as it happened in the CRS patient we treated.

The formation of the vertebral column during embryogenesis is a critical process termed somitogenesis [27]. This process begins when somites bud off from the anterior tip of the paraxial mesoderm, presomitic mesoderm (PSM), following a sequential organization along the anterior-posterior axis of the embryo [27]. Once the PSM has been formed, a rhythmic formation of somites begins. This has been postulated to be dependent on the activation of three signaling pathways; Notch, Wnt/β-catenin, and fibroblast growth factor (FGF). As a result of this activation, and that of other specific signaling circuits, a progressive wave of gene expression appears along the embryonic axis. Among genes that are expressed cyclically, the *hairy1* gene, regulated by Notch, seems to play a key role. The expression of this gene follows a dynamic pattern, first expressed in the caudal region and then moving to the anterior region as a new somite is formed. Therefore, the somitogenesis occurs as a result of the periodic activity, regulated in time of formation and number of formed somites, known as the “segmentation clock”, which is specific to each species. This “clock” is then responsible for the progressive and symmetrical bilateral formation of the somites during neurulation [27]. The specificity of somites along the antero-posterior axis is established by the expression of the homeobox gene *Hox*, activated by the “segmentation clock” during the formation of each somite. This allows the coordination between the formation and the anatomical specification of each new segment. However, the specificity of each somite only takes place when it has maturated completely. Then, from the somite cells closest to the neural tube, vertebrae and ribs will be formed; from the somite cells located in the more dorsal region, the dorsal dermis (dermatome) will be formed, while the motor equivalent to dermatome, termed myotome, proceeds from the cells present at the borders of each somite that give rise to the skeletal musculature. As described, somites are the origin of vertebrae, dorsal dermis, and muscles but also of nerves, blood vessels, and connective tissues such as tendons and ligaments [28]. This evolution into different tissues is strictly and specifically controlled, depending on a number of transcription factors; Pax1 (vertebrae), neurotrophin 3 and Wnt1 (dermatome), Wnt1, Wnt3a, BMP4, FGF5, and Wnt (myotome), the later three bring expressed by the neural tube and by the lateral mesoderm. Similarly, the limits of somite formation, the point at which the activity of the “segmentation clock” ends, are strictly controlled. Basically, this seems to depend on the existence of a gradient between FGF8 (androgen-induced growth factor 8) and WTN signaling along the rostrocaudal PSM axis, which decreases rostrally from the caudal PSM, although many other factors participate in this process. Although this mechanism for the formation of somites, and therefore for the axial segmentation, is widely accepted, it has been proposed recently that somites are able to self-organize, controlled by local cell–cell interactions, without needing any segmentation clock or a gradient of specific factors [29]. According to the data of these authors, treatment with Noggin non-somite mesoderm leads to the simultaneous formation of many normal somites that also possess axial identity. However, these somites cannot form the rostral and caudal halves needed for neural segmentation [29].

More recently, a novel role in the process of segmentation and, consequently, in the pathogenesis of vertebral abnormalities has been described for Golgi proteinase, a member of the family of serine proteases, MBTPS1/SKI-1/S1P (membrane bound transcription factor protease 1, Subtilisin/kexin isozyme-1 or, as previously termed, Site 1 protease). The loss of the function of the *Mbtps* 1 gene, or its deletion during embryogenesis, leads, in mice, to the appearance of phenotypic changes localized in the lumbosacral vertebral region that mimic those observed in CRS. Therefore, the *Mbtps1* gene plays critical roles in regulating somatogenesis [28].

Although it was not the objective of this work, given how critical the development of the vertebral column is, it is easy to understand that any single alteration during this period may lead to the appearance of many different abnormalities. Moreover, since this stage of the embryonic development occurs sequentially, depending on when the alteration has affected somitogenesis, the severity of the resulting abnormalities may be different. In addition, since somitogenesis occurs during a restricted period of time during embryogenesis, we can also understand why GH administration did not induce any positive change in the abnormal vertebral column of the patient we treated, despite the close relationships between GH, FGF, and Notch [6].

Years ago it was postulated that the GH/IGF-I axis plays a significant role in neural development and neural injuries [30]. Some years before, it had been found that the GH receptor (GHR) was expressed in areas of the brain in which the formation of neurons occurs during the development of the embryonic brain [31]. Following studies revealed that GH could be found in the cells of brain neurogenic niches, such as the sub-ventricular zone, during neurogenesis in the embryo [32]. Moreover, postnatally the hormone is produced in the hippocampus [33,34,35,36]. The role of brain GH is related to the induction of proliferation and differentiation of neural stem cells (NSCs), both observed in cultures of brain cortical zones of embryonic rats [37] and hippocampus of aged mice [35,36,38]. However, not only the locally expressed GH plays a role on embryonic and adult neurogenesis, but also the GH administered exogenously and the pituitary GH itself contribute to this process, even in humans. In fact, the administration of GH to NSCs obtained from fetal human forebrains induce the proliferation and migration of these cells [39], a finding also observed for prolactin (PRL) [39]. While the effects of locally produced GH seem to play a physiological role, it cannot be discarded that the local production of the hormone also acts therapeutically after a brain injury. It would be interesting to analyze whether GH is overexpressed in the cerebral neurogenic niches after damage. However, its ability for promoting brain repair has to be dependent on the magnitude of the damage incurred. This can be compensated with the administration of exogenous GH, as we demonstrated in rats [40], in which a brain injury induced by kainate administration led to an increase in the proliferation of hippocampal NSCs, which were significantly higher when GH was given [40], indicating that exogenous GH had cooperated with the locally produced hormone. Moreover, even in the brain of adult rats without any damage, the hormone induces similar proliferative effects [41].

Given the known positive effects of GH on the brain [6], we and others obtained significant improvements in patients with many different brain injuries occurring at different ages, treating them with GH and specific rehabilitation [7,8,10,11,42,43,44].

Less known are the effects of GH in the SC, but it seems to be logical that the hormone may act there as it does in the brain. The SC ependyma holds a neurogenic potential [45], and a number of Nestin (a marker of neural progenitor differentiation towards neurons) immunoreactive cells has been detected at all three SC levels in humans who died after an accident or nontraumatic causes [46]. This suggests that in the SC there is a population of neural progenitor cells with the potential for proliferation, differentiation, and migration, as occurs in the brain. These SC NSCs have been proposed to play a protective role after a SC injury, decreasing the loss of tissue induced by the injury [47], and potentially representing a source for repairing SC injuries [46,48]. In fact, after a SC injury in rats, NSC activity appears in SC ependymal cells [49]. However, a recent study in normal human adults and patients with SC injuries, questions that the SC ependyma may have neurogenic properties, at least in adult humans (beyond the age 18 years) [50]; rather, these authors suggest that the adult SC ependymal region resembles more a low-grade ependymoma than a neurogenic niche. This, however, does not apply to children, as the authors affirm in their study [50].

A study of transgenic mice expressing a growth hormone antagonist demonstrated that, after birth, the neural effects of the hormone are more evident in the SC than in the brain and that the SC continues to show GH dependence into adulthood [51]. There is GH and GHR immunoreactivity in the embryonic SC of chickens [52], and almost 20 years ago it was demonstrated that transgenic mice overexpressing GH showed an increase in the size of lumbar SC motoneurons parallel to the increase observed in their body size, in comparison to their non-transgenic littermate controls [53]. More recently, it has been shown that the amount and enzymatic activity of acetylcholinesterase was notably diminished in the SC of GH-deficient rats; since this enzyme is a marker for cholinergic neurons and their synapses, these data suggest that these findings are due to the lack of positive effects of GH on the proliferation of cholinergic synapses in the rats’ SCs [54]. Moreover, topical application of GH or nanowired delivery of the hormone to a rat model of injured SC promotes neuroprotection, decreasing the degree of edema formation and neuronal SC injuries [55]. In addition, GH is expressed in the peripheral nervous system [56].

Taken together, these data indicate that GH may act in the SC as it does in the brain [6]. This would explain the results we obtained in this case of CRS. That is, the administration of GH may have led to the increased proliferation and differentiation of ependymal stem cells, allowing the formation of the neural components responsible for the development of tje new innervation (sensitive and motor) observed. Once GH provided the needed nerve support, rehabilitation improved the functional significance of afferent and efferent nerve pathways. Since the SC did not grow below the level of its interruption (see Figure 3 and Figure 4), we only can explain the innervation produced in the patient because new and specific spinal nerves have formed from L2, due to the action of GH on the SC ependymal stem cells. Another possibility, more feasible, is that from the last existing spinal nerve a net of new specific connections had been formed. Most likely a Diffusion Tensor MRI (tractography) study would give the right answer, but it still could not be done. While these explanations are merely speculative, the real thing is that the patient completely acquired innervation that did not exist. This cannot occur spontaneously in this syndrome or after specific intensive rehabilitation.

Given that the patient lacks the sacrum bone and his hips are luxated, we are now trying to develop an artificial sacrum, made in the laboratory with a decellularized matrix provided from a dead donnor and autologous mesenchymal stem cells expanded in GMP conditions. Theoretically, the implant of this sacrum would allow it to grow as the patient grows. Before doing this, we will study in rats whether this is possible. Another option would be to implant an artificial sacrum which would be able to grow by means of external screws manipulated by therapists after radiological controls had been done. Apart from this, the patient will need a very complex surgery to reconstruct the hips, detethering the SC, and he will need to be carefully controlled for a possible increase of hydrosyringomyelia.

## 4. Materials and Methods

The patient was a 9-month-old male born of a nonconsanguineal marriage by scheduled caesarean that presented with caudal regression syndrome with sacral agenesis (detected in utero by ultrasonography), right renal agenesis, left hydronephrosis, neurogenic bladder and bowel, tne absence of innervation (sensitive and motor) of legs, scoliosis, passive knees flexion, and clubfoot. Apgar score at birth was 3/4 (1 min/5 min) and the pH in blood cord was 7.3; his weight at birth was 2.634 kg (p10) and his size was 41 cm (<p10). General conditions were very bad at birth and the patient had to be reanimated (oro-tracheal intubation).

The patient was the first and unique child of a non-diabetic woman; his weight was normal for the gestational age. The mother did not have any kind of toxic habits (alcohol, tobacco, drugs); she did not take any kind of pharmaceutical drugs during pregnancy, she was not exposed to toxic agents, organic fat solvents, or radiation, and no remarkable incidents occurred during gestation.

An X-ray exam of the vertebral column performed at day 1 of age showed agenesis of the sacrum and L5 vertebra and that the hypoplastic portion of L4 was articulated with iliac pseudoartrosis. These correspond with a sacral agenesis Type IV, according to the Renshaw classification of this developmental abnormality [17].

To correct clubfoot, his feet were plastered at age 4-days. The patient suffered multiple urine infections and a cystography carried out 2-days after birth detected passive vesicoureteral reflux to the whole excretory way of the left kidney. At age 7-days, an MRI confirmed the lack of development of the vertebral column, which stopped in L3 (Figure 4), as well as the existence of sacral agenesis and a hypertense signal of fat intensity most likely corresponding to a lipoma.

An electromyogram revealed a slight innervation of the psoas and quadriceps, with complete denervation of any other muscle of the legs and feet (data not shown).

Molecular exams (Multiplex ligation-probe amplification; MRC-Holland) carried out in his hospital of reference did not detect any alteration in the subtelomeric regions analyzed; the karyotype was that of a normal male (46, XY) and the analysis of multiple genes that might be involved in the appearance of the syndrome was also normal.

From early after birth, he received rehabilitation in his hospital of reference (Vojta method therapy).

At admission to our center (age 9 months), the patient had a height slightly below p3 for his age, mainly due to the marked hypotrophism of his legs, while his weight was in p50. The physical examination revealed a high hypotony in his legs (his thighs were practically composed by fatty tissue, without detectable muscular tissue) and fully paralytic and hypotrophic clubfoot. His knees were in an irreductible passive flexion of 68° and 80° (right and left knees respectively), as measured with a goniometer. There were important retractions in the pelvic musculature, mainly in the hip flexors. The pelvic diameter was reduced because of an articulation of the iliac bones. There was luxation of both hips. Plantar flexion and foot dorsiflexion did not exist.

The Gross Motor Function Test (GMFM-88) and Modified Ashworth Scale were carried out before the start of treatment, in some of the controls carried out during it, and five years after the treatment commenced. In parallel to these tests, the patient was also examined according to the sensory and motor standards for the neurological classification of SC injury established by the American Spinal Cord Injury Association (ASIA).

No responses existed to any kind of sensitive stimulation of the buttocks and legs. There was an almost continuous flow of urine and liquid feces. The child was able to maintain sedestation, but unable to perform a normal crawl, since he only utilized his arms (Movie 1).

After the physical examination, the Battelle Developmental Inventory Screening test (BDIST) was performed. This test screens and evaluates early childhood developmental milestones. Results from this evaluation indicated that no cognitive stimulation was needed, because the scores reached in each of the areas explored were normal or higher than those expected for the age of the patient, particularly at the cognitive level. The only exception was the motor area, a logical result given the pathology of the patient.

Routine blood analysis (hematimetry and biochemistry) and the analysis of some important hormones (plasma TSH, fT4, morning cortisol, IGF-I, and IGFBP3) were carried out before the treatment with GH, at 3-month intervals during the first year of it, and, later, every 6-months.

Studies and treatments were conducted according to our protocols and in compliance with the Spanish legislation for using GH “off label” and the Code of Ethics of the World Medical Association (Declaration of Helsinki). Signed informed consent for using GH and melatonin was obtained from the legal representatives of the patient. The rehabilitation consisted of daily physical therapy (2 h/day, 5 days/week). Once the patient was growing and significant sensitive and motor improvements were observed, a session of pelvic floor therapy was added (1 h/week) to the two daily sessions of physiotherapy.

GH treatment started in parallel with physiotherapy. Initially, GH (Nutropin, Ipsen) was given at a daily dose of 0.3 mg (5 days/week) for 3-months, followed by 15 days resting. After this, the dose was increased to 0.5 mg/day, following the same schedule. The rationale for this kind of GH treatment was based on the fact that we attempted to stimulate both the proliferation and differentiation of NSCs existing in the SC (ependymal stem cells) and not to induce the longitudinal growth of the organism. It has been reported that high concentrations of GH reduce NSC proliferation and promote their differentiation [37,57]. Conversely, treatment with GH doses, which stimulate neural stem cell proliferation, significantly reduced neuronal differentiation [32,58]. Moreover, NSCs deplete over time after being continuously activated [59]. That is, we did not treat a possible GH-deficiency, but tried to achieve better NSC responses to GH administration.

During the first stage the patient remained in treatment at the Medical Center Foltra for a period of seven months. After this, and because of work problems experienced by his parents, he was discharged from our center and referred to another center of physiotherapy closer to his home; there the same treatment procedures were followed, as indicated by us. Every 3-months he came back to the Medical Center Foltra for an assessment of his evolution, and new instructions for rehabilitation, or increasing GH doses were given if it was appropriate. One year later, the patient came back to our center for a second stage of 6-months; after this, and given his good evolution, he was discharged and physiotherapy was carried out at home by his parents. GH administration and physical and analytical controls have continued until now. Currently the patient is 6-years old and the GH dose is 1 mg/day. The last movie (Movie 7), recorded a few days ago, shows that now the patient is able to reach bipedestation by himself and maintain this position for some time.

Due to the existence of hydronephrosis in his unique kidney, renal function was periodically controlled by a nephrologist from his hospital of reference.

Videos were taped in Medical Center Foltra and by the parents of the patient. The parents of the patient gave signed informed consent for the publication of the images and videotapes of the patient.

## 5. Conclusions

Our data indicate that a treatment with GH and rehabilitation in early stages of life seems to be useful for acquiring innervation of distal SC segments previously lacking it and for improving the quality of life of some cases of CRS, hitherto considered as only being able to receive supportive measures.

## Supplementary Materials

Supplementary materials can be found at www.mdpi.com/1422-0067/18/1/230/s1.
