# Supplementary material for: Growth Hormone (GH) and Rehabilitation Promoted Distal Innervation in a Child Affected by Caudal Regression Syndrome
Source: Int J Mol Sci. 2017 Jan 23;18(1):230. doi: 10.3390/ijms18010230 (PMC5297859; doi:10.3390/ijms18010230)
Supplement: Supplementary file 1 [file ijms-18-00230-s001.zip › movie legend.pdf]

Movie 1. The video shows the patient at age 1-year old during a session of physiotherapy in the Medical Center Foltra, after 3-months of treatment with GH and rehabilitation. As shown in the video, the inferior limbs are fully paralytic and clubfoot can be seen.

Movie 2. After 2-years of treatment full sensitivity exists in the inferior limbs.

Movie 3. 2-years since the treatment commenced. The patient began to walk with a walker during a session of physiotherapy in the Medical Center Foltra.

Movie 4. 2-years of treatment. The patient was able to ride in tricycle, both forward and backward.

Movie 5. 5-years of treatment; the patient was able to make plantar flexion against resistance. A weak dorsiflexion can also be observed.

Movie 6. It shows the motor evolution of the patient, since 8-months after he commenced the treatment. The video shows how his muscles are increasing their volume and they are going to be able for doing voluntary contractions. It can be seen too the difference between the right and the left legs and feet.

Movie 7. The patient at age 6-years. He is now able to standing up and maintaining the bipedestation for a timw without any help.
